# Chemokines in Cancer Development and Progression and Their Potential as Targeting Molecules for Cancer Treatment

**DOI:** 10.1155/2014/170381

**Published:** 2014-05-22

**Authors:** Naofumi Mukaida, So-ichiro Sasaki, Tomohisa Baba

**Affiliations:** ^1^Division of Molecular Bioregulation, Cancer Research Institute, Kanazawa University, Kakuma-machi, Kanazawa 920-1192, Japan; ^2^Japan Science and Technology Agency, Core Research for Evolutional Science and Technology, Chiyoda-ku, Tokyo 102-0075, Japan

## Abstract

Chemokines were initially identified as bioactive substances, which control the trafficking of inflammatory cells including granulocytes and monocytes/macrophages. Moreover, chemokines have profound impacts on other types of cells associated with inflammatory responses, such as endothelial cells and fibroblasts. These observations would implicate chemokines as master regulators in various inflammatory responses. Subsequent studies have further revealed that chemokines can regulate the movement of a wide variety of immune cells including lymphocytes, natural killer cells, and dendritic cells in both physiological and pathological conditions. These features endow chemokines with crucial roles in immune responses. Furthermore, increasing evidence points to the vital effects of several chemokines on the proliferative and invasive properties of cancer cells. It is widely acknowledged that cancer develops and progresses to invade and metastasize in continuous interaction with noncancerous cells present in cancer tissues, such as macrophages, lymphocytes, fibroblasts, and endothelial cells. The capacity of chemokines to regulate both cancerous and noncancerous cells highlights their crucial roles in cancer development and progression. Here, we will discuss the roles of chemokines in carcinogenesis and the possibility of chemokine targeting therapy for the treatment of cancer.

## 1. Introduction

Chemokines are heparin-binding proteins with 4 cysteine residues in the conserved positions [1]. Two intermolecular disulfide bonds are formed between the first and third cysteines and between the second and fourth cysteines. These bonds lead to the formation of triple-stranded *β*-sheet structures, while the carboxyl-terminal region forms a *α*-helix form [2]. This accounts for their similar three-dimensional structure despite their low overall sequence similarities. Chemokines exert their biological activities by binding their corresponding receptors, which belong to G-protein coupled receptor (GPCR) with 7-span transmembrane portions [1]. Thus, the target cell specificity of each chemokine is determined by the expression pattern of its cognate receptor (Table 1). Moreover, chemokines can bind to proteoglycans and glycosaminoglycans with a high avidity, because the carboxyl-terminal region is capable of binding heparin. Consequently, most chemokines are produced as secretory proteins, but upon their secretion, they are immobilized on endothelium cells and/or in extracellular matrix by interacting with proteoglycans and glycosaminoglycans [2]. The immobilization facilitates the generation of a concentration gradient, which is important for inducing the target cells to migrate in a directed way.

Chemokines are structurally divided into 4 subgroups, namely, CXC, CC, CX3C, and C [1]. The first 2 cysteines are separated by 1 and 3 amino acids in CXC and CX3C chemokines, respectively, while the first 2 cysteines are adjacent in CC chemokines. The C chemokines lacks the second and fourth cysteines [1]. The CXC chemokines are further grouped based on the presence or the absence of a 3-amino acid sequence, glutamic acid-leucine-arginine (the ELR motif), immediately preceding the CXC sequence [3]. Chemokines can be functionally classified as inflammatory, homeostatic, or both, based on their expression patterns [4]. Various types of inflammatory stimuli induce abundantly the expression of inflammatory chemokines to induce the infiltration of inflammatory cells such as granulocytes and monocytes/macrophages. Representative inflammatory chemokines are CXC chemokines with ELR motif and CCL2. On the contrary, homeostatic chemokines are expressed constitutively in specific tissues or cells. They have a crucial role in organogenesis of various organs including lymph nodes, arising from their key roles in stem cell migration. Moreover, most homeostatic chemokines can control the movement of lymphocytes and dendritic cells and eventually adaptive immunity.

The human and mouse genomes contain over 44 and 38 different chemokine genes, respectively [5]. There is a difference in gene numbers with some ambiguities of orthologous relationship between the human and mouse chemokine family. These observations would indicate species-specific expansions and contractions in chemokine genes, resulting from their rapid evolution. A prominent difference is found in one major chemokine, CXCL8, and its receptors, CXCR1 and CXCR2. Mice and rats do not possess a homolog of the* CXCL8/IL-8* gene, which is present in other species including humans, rabbits, cats, and dogs [5]. Moreover, the CXCR1 and CXCR2 genes encode functional receptor proteins in humans, whereas there still remains a question on the presence of functional CXCR1 gene in mice or rats [6]. Furthermore, humans and mice exhibit different expression patterns also in other chemokine receptors such as CCR1 [7]. These observations should be taken into consideration when the findings obtained with mouse models are extrapolated into human conditions.

Here, we will review the potential roles of chemokines in tumor development and progression by focusing on their effects on noncancerous and cancerous cells. We will further discuss the potential of chemokine targeting therapy for cancer treatment.

## 2. Effects on Noncancerous Cells

### 2.1. Leukocytes

Since the first description by Virchow more than a century ago, it is widely acknowledged that leukocytes are present in both the tumor areas and the tumor-supporting stroma [8]. Moreover, leukocytes might account for up to 50% of the tumor mass, the most predominant subset being macrophages. Tumor-associated macrophages (TAMs) are derived mostly from circulating monocytes which are attracted into tumor sites by locally produced chemotactic factors, such as CCL2, CCL5, CCL7, CCL8, and CXCL12, and macrophage colony stimulating factor (M-CSF) [8]. Among these chemotactic factors, CCL2 is presumed to play an important role in TAM recruitment [8, 9]. Repeated dextran sodium sulfate (DSS) solution ingestion causes the development of multiple colonic tumors in mice, which received a prior administration of azoxymethane (AOM). The resultant colonic tumors contain a large number of monocytes/macrophages expressing cyclooxygenase (COX)-2, an enzyme crucially involved in colon carcinogenesis [10]. Abundant CCL2 is detected in colon tissues, and CCL2 blockade reduces the infiltration of CCR2-positive COX-2 expressing monocytes/macrophages and eventually colonic tumor development and progression [10].

**Figure 1:**
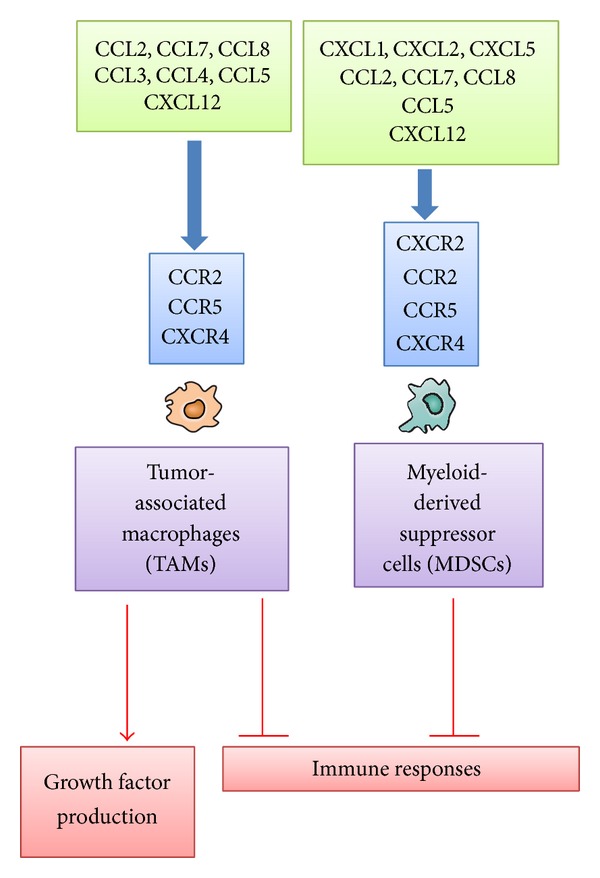
Chemokines acting on TAMs and MDSCs.

TAMs produce various growth factors such as vascular endothelial growth factor (VEGF) and fibroblast growth factor (FGF) in addition to prostaglandin [8, 9]. Monocytes are recruited by CCL2 to pulmonary metastatic sites of murine breast cancer and promote the extravasation of tumor cells, a necessary step for metastasis, in a process that requires monocyte-derived VEGF [11]. Moreover, TAMs exhibit the properties of M2-polarized macrophages and are capable of producing immunosuppressive molecules including IL-10, transforming growth factor (TGF)-*β*, and arginase [12]. These properties endow TAMs with an immunosuppressive capacity. Thus, TAMs can promote tumor progression through the production of growth factors and the suppression of antitumor immunity (Figure 1).

Tumor tissues contain myeloid-derived suppressor cells (MDSCs), which can suppress adaptive immunity. MDSCs are characterized by the coexpression of the myeloid-cell lineage differentiation antigen Gr-1 and CD11b in mice, while they are defined as CD14^−^CD11b^+^ cells or as cells that express the common myeloid marker CD33 but lack the expression of mature myeloid and lymphoid markers in humans [13]. MDSCs contain abundantly immunosuppressive enzymes, arginase 1 and inducible NO synthetase (iNOS), and produce immunosuppressive cytokines such as TGF-*β*1 and IL-10, thereby inhibiting the T-cell response [13]. CCL2 recruits MDSCs in several types of mouse cancer, including Lewis lung carcinoma, meth A sarcoma, melanoma, and lymphoma [14]. However, CCR2 deficiency results in the conversion of the MDSC phenotype from macrophage lineage to neutrophil lineage without affecting tumor growth [15]. MDSCs, particularly granulocytic ones, express CXCR2 and are plentifully present in colonic tumors developed after the combined treatment of AOM and DSS [16]. CXCR2 ligands, such as CXCL1, CXCL2, and CXCL5, are present abundantly in the colon tumor tissues and the loss of CXCR2 dramatically suppresses tumorigenesis through inhibiting MDSC infiltration [16] (Figure 1).

**Figure 2:**
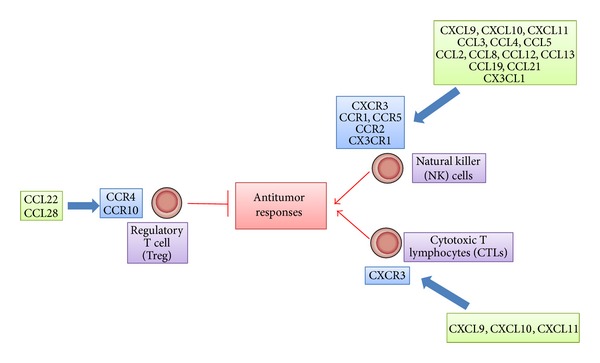
Effects of chemokines on antitumor responses.

Regulatory T (Treg) cells have a crucial role in the maintenance of immunological self-tolerance [17]. A large number of Treg cells often infiltrate into tumors and systemic removal of Treg cells enhances natural as well as vaccine-induced antitumor T-cell immunity. Treg cells express CCR4, and its ligand, CCL22, regulates intratumoral Treg infiltration in various tumors [17]. Hypoxia induces the expression of another chemokine, CCL28, in tumor sites. CCL28 promotes angiogenesis and recruits Treg cells, thereby also propagating immune tolerance [18] (Figure 2).

Bindea and colleagues demonstrated that T follicular helper (Tfh) cells and B cells infiltrated tumor sites of human colorectal cancer patients, along with tumor progression [19]. Moreover, the numbers of B cells were associated with prolonged survival. Furthermore, when colon cancer cells were endoscopically injected into the colon submucosa, CXCL13 injection reduced tumor formation, whereas the deficiency in* CXCR5* gene, a receptor for CXCL13, accentuated tumor formation [19]. Thus, the CXCL13/CXCR5 axis might be pivotal factors for the Tfh/B cell infiltration into tumor sites and subsequent tumor formation.

**Figure 3:**
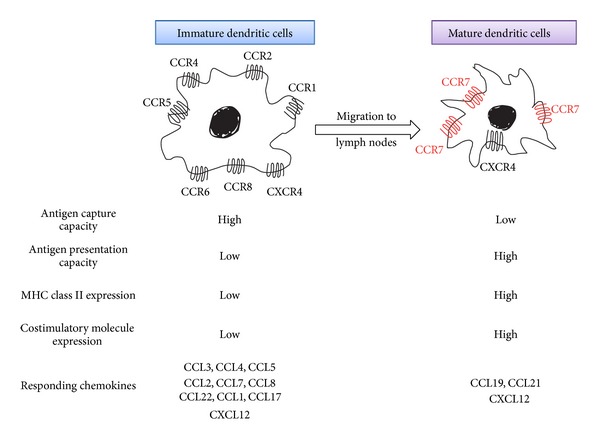
Chemokines acting on dendritic cells at different maturation stages.

Antitumor responses are attributable to tumor infiltrating lymphocytes (TILs), particularly cytotoxic T lymphocytes (CTLs) [20]. CTLs can specifically recognize tumor-associated antigens (TAAs) and attack tumor cells in humans as well as in mice [21] (Figure 2). In this process, antigen-presenting cells can deliver TAAs and prime TAA-specific T cells. Dendritic cells (DCs) are professional antigen-presenting cells and can express on their cell surface major histocompatibility complex (MHC) class I and II molecules and costimulatory molecules, all of which assist in T-cell activation [21]. Immature DCs are distributed in almost every peripheral tissue and express several chemokine receptors including CCR1, CCR2, CCR4, CCR5, CCR6, CCR8, and CXCR4, with a high capacity to endocytose various materials [22]. DCs capture exogenous and endogenous antigens including tumor cell-derived antigens in periphery (Figure 3). When DCs capture antigens in the presence of inflammatory stimuli such as toll-like receptor-mediated signals, they change to a mature state and lose endocytosis ability. They start to express a limited set of chemokine receptors, CCR7 and CXCR4, and migrate into the T-cell areas of regional lymph nodes via afferent lymphatic venules under the guidance of chemokines [23] (Figure 3). Indeed, the appearance of apoptotic tumor cells induces the migration of dendritic cells to the draining lymph nodes and eventually generates a specific cytotoxic T lymphocyte population in the draining lymph nodes by utilizing the CCL3-CCR5/CCR1 axis [24]. On the contrary, DCs fail to express costimulatory molecules and to present antigens efficiently if they capture antigens in the absence of inflammatory cues. Mature DCs exhibit enhanced expression of costimulatory molecules and process the antigens into the peptides. The resultant peptides are presented to T cells in conjunction with MHC molecules, in the regional lymph nodes, to induce primary immune responses [23] (Figure 3).

Once generated in the regional lymph nodes, TAA-specific CTLs should migrate to tumor sites to kill tumor cells. Numerous clinical studies have indicated that the intratumoral presence of CD3^+^ or CD8^+^ lymphocytes has a positive prognostic influence on survival [25]. Several chemokines can regulate the migration of TILs, particularly CTLs, into tumor sites. CXCR3 is deemed to be a major chemokine receptor expressed by TILs. In a mouse model, increased expression of ligands for CXCR3, CXCL9, and CXCL10 can elicit antitumor response accompanied with an enhanced infiltration of CD4^+^ and CD8^+^ lymphocytes [26]. Consistently with this observation, in human gastric and colorectal cancer, TILs express CXCR3 (Figure 2) [27–29]. Moreover, high levels of CXCL9 and CXCL10 are produced by stromal cells, mainly macrophages [28]. CD8^+^ TILs also express another chemokine receptor, CCR5 [27, 29]. Concomitantly, CD8^+^ TIL numbers correlate well with the expression of CCL5, a ligand for CCR5, by tumor tissues [29]. TILs express additional chemokine receptor, CX3CR1, and the expression of its ligand, CX3CL1, is elevated in tumor cells in colorectal cancer tissues [30]. Furthermore, the expression level of CXCL16 also correlates with CD4^+^ and CD8^+^ TIL as well as numbers with a better prognosis although cells expressing CXCR6, a receptor for CXCL16, are not identified [31]. Thus, CXCL9, CXCL10, CXCL16, CCL5, and CX3CL1 can be used to efficiently mobilize CTLs from regional lymph nodes to tumor tissues with an objective to enhance CTL-mediated tumor destruction.

Natural killer (NK) cells are unconventional lymphocytes, which can* in vitro* kill a broad range of tumor cells of both hematopoietic and nonhematopoietic origin by utilizing perforin and secreting interferon (IFN)-*γ* [32]. Moreover,* in vivo*, mouse NK cells can eliminate many transplantable and spontaneous tumors. NK cells express distinct sets of chemokine receptors (Table 1). NK cells migrate to lymph nodes mainly by utilizing CXCR3, while their migration to the inflamed tissues including tumor sites involves CCR1, CCR2, CCR5, CXCR3, and CX3CR1 (Figure 2) [33]. Thus, the ligands for these receptors can regulate NK cell trafficking and augment their functions. However, in colorectal tumor tissues, NK cells are scarce despite a significant lymphocyte infiltration, even in the presence of high levels of CXCL9, CXL10, CCL3, CCL4, CCL5, and CX3CL1 [34]. Thus, NK cell migration into tumor tissues may be impaired early in the course of tumor development by the mechanism that does not affect CTL trafficking.

We recently succeeded in causing chronic myelogenous leukemia (CML)- like pathology by direct transplantation of* BCR-ABL* gene-transduced leukemia initiating cells (LICs) into the bone marrow cavity of nonirradiated mice [35]. We further observed that BCR-ABL^+^lineage^−^c-kit^−^ immature leukemia cells produced high levels of CCL3, which promoted the development of CML. Conversely, the ablation of* CCL3* gene in LICs dramatically inhibited the development of CML and concomitantly reduced recurrence after the cessation of a short-term tyrosine kinase inhibitor treatment. Moreover, normal hematopoietic stem/progenitor cells (HSPCs) can directly impede the maintenance of LICs in bone marrow in the absence of CCL3 signal. These observations would indicate that leukemia cell-derived CCL3 expels normal HSPCs from bone marrow to make spaces for leukemia cells to survive [35].

### 2.2. Endothelial Cells

**Figure 4:**
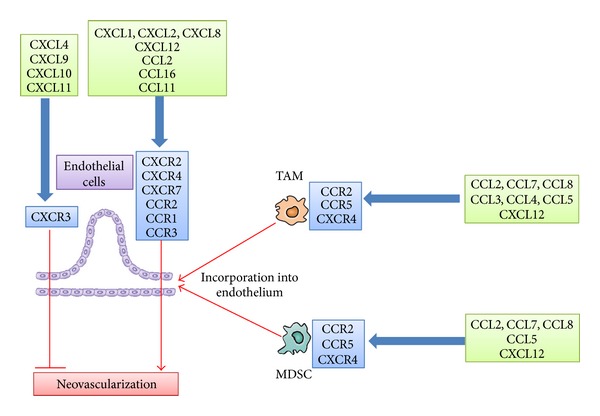
Chemokines acting on tumor neovascularization.

Neovascularization is crucial for tumor growth, progression, and metastasis [36]. The ELR-motif-positive CXC chemokines, CXCL1, CXCL2, CXCL3, CXCL5, CXCL6, CXCL7, and CXCL8, directly promote the migration and proliferation of endothelial cells and eventually neovascularization, mainly by interacting with CXCR2 but not with CXCR1 [37]. CXCL12 is not an ELR-positive CXC chemokine but has a potent angiogenic activity [38]. Three CC chemokines, CCL2, CCL11, and CCL16, have also been implicated in tumor neovascularization [39–41]. CCR2, a specific receptor for CCL2, is expressed by endothelial cells and CCL2 exerts its angiogenic activity in a membrane type 1- (MT1-) matrix metalloproteinase- (MMP-) dependent manner [39] (Figure 4).

CXCL4 and interferon-inducible ELR-negative CXC chemokines such as CXCL9, CXCL10, and CXCL11 inhibit the angiogenesis induced by ELR-motif-positive CXC chemokines, VEGF, and bFGF [42, 43] by interacting with a common receptor, CXCR3 (Figure 4). Moreover, the Duffy antigen has been shown to suppress the angiogenic effects of the ELR-motif-positive CXC chemokines, as it can sequester the ELR-motif-positive CXC chemokines without eliciting any intracellular signals [44]. Thus, the balance between proangiogenic and antiangiogenic chemokines may determine the degree of tumor neovascularization.

TAMs and MDSCs contributed to tumor angiogenesis by producing a wide variety of angiogenic factors such as VEGF, TGF-*β*, CXCL8, platelet-derived growth factor (PDGF), and MMPs such as MMP-2 and MMP-9 [8, 9]. Several chemokines, particularly CCL2, can induce tumor angiogenesis by attracting TAMs and MDSCs. Moreover, recruited TAMs and MDSCs can acquire endothelial cell phenotypes and be incorporated into the newly formed vascular structure (Figure 4) [45]. Endothelial cell-derived ELR-motif-positive CXC chemokines, especially CXCL6, induce angiogenesis in gastrointestinal cancer by recruiting neutrophils [46].

### 2.3. Fibroblasts and Other Cells

Fibroblasts present in tumor tissues are designated as cancer-associated fibroblasts (CAFs). CAFs can produce tumor-promoting molecules such as TGF-*β*, FGF, hepatocyte growth factor (HGF), and EGF [47]. The proposed cellular sources of CAFs include locally resident fibroblasts, cells undergoing epithelial-mesenchymal transition (EMT), cells undergoing endothelial-mesenchymal transition (EndMT), or bone marrow- (BM-) derived mesenchymal stem cells (MSCs) [48]. In a mouse gastric cancer model, it is estimated that BM-derived MSCs can contribute as much as 25% to the CAF population [49]. On the contrary, in a mouse lung metastasis model, BM-derived cells do not significantly contribute to fibroblast accumulation in lungs [50].

In a mouse gastric cancer model, CXCL12 expression was found to be enhanced together with enhanced fibrosis in tumor sites [49]. Because MSCs express CXCR4 and CXCR7 and migrate to their ligand, CXCL12, the CXC12-CXCR4/CXCR7 axis may regulate the accumulation of fibroblasts and consequent fibrosis development [48]. Moreover, CAFs present in various types of cancer produce CXCL12, and the produced CXCL12 promotes the angiogenic, proliferative, and migratory properties of tumor cells. CAFs produce CCL2, CCL5, CCL7, CXCL8, and CXCL14 and these chemokines promote tumor progression mainly by enhancing the motility of tumor cells [48].

In a mouse lung metastasis model, HGF-expressing fibroblasts were found to be increased in the lungs [50]. Lung fibroblasts express CCR5, and genetic deletion of CCR5 or its ligand, CCL3, attenuates intrapulmonary lung metastasis formation along with reduced fibroblast accumulation and HGF expression [50]. We recently observed that fibroblast accumulation was required for full-blown progression of chronic colitis-associated colon cancer, in addition to inflammatory cell infiltration. Moreover, fibroblast accumulation in colon tissues was regulated by the CCL3-CCR5 axis.

When activated, hepatic stellate cells (HSCs) express a marker of myofibroblasts, *α*-smooth muscle actin (*α*-SMA). *α*-SMA-positive HSCs secrete CXCL12 similarly as CAFs do [51]. Moreover, in a mouse liver metastasis model, tumor-derived CCL2 was found to induce *α*-SMA-positive HSCs to accumulate at tumor sites and to express MMP-2. Genetic ablation of CCR2 markedly attenuates tumor formation with reduced HSC accumulation and MMP-2 expression [52]. Thus, CCR2-mediated signals may regulate the trafficking and functions of HSCs, thereby inducing liver metastasis.

## 3. Direct Effects on Cancerous Cells

The tyrosine kinase, RET, is a prototypic transforming oncogene in human papillary thyroid carcinoma and induces the expression of several chemokines including CCL2, CCL20, ELR-motif-positive CXC chemokines, and CXCL12, and CXCR4 [53]. The* CXCR4* gene can further be transactivated by the activation of hypoxia-inducible factor- (HIF-) 1*α*, arising either from loss of the von-Hippel-Lindau tumor suppressor (VHL) or due to the hypoxic conditions observed frequently in tumor tissues [54]. Moreover, components of the Ras-Raf signaling pathway can activate NF-*κ*B [55], which can lead to enhanced expression of chemokines, including CXCL1, CXCL8, and CCL2 [56]. Thus, even in the absence of direct oncogene activation, NF-*κ*B and HIF activation can induce the expression of chemokines and their receptors, in tumor tissues.

**Figure 5:**
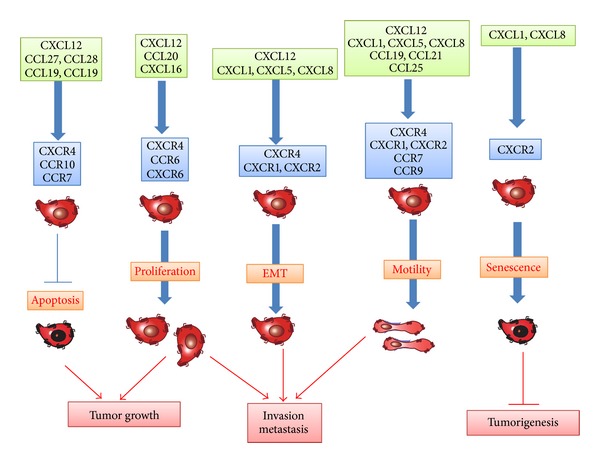
Effects of chemokines on tumor cells.

The term “cellular senescence” has been used to denote a stable and long-term loss of proliferative capacity, despite continued viability and metabolic activity [57]. Activation of oncogenes, particularly Ras, induces cellular senescence which is designated as oncogene-induced senescence (OIS). OIS serves as a potent barrier against oncogenic transformation by suppressing the unscheduled proliferation of early neoplastic cells [57]. Moreover, cells undergoing OIS secrete CXCR2-binding chemokines and IL-6 through the activation of 2 proinflammatory transcription factors, C/EBP-*β* and NF-*κ*B [58, 59]. CXCL8/IL-8 specifically colocalizes with arrested p16^INK4A^-positive epithelium in human colon adenomas [58]. Furthermore, the reduction of CXCR2 expression on tumor cells alleviates OIS and diminishes the DNA-damage response, while ectopic expression of CXCR2 results in premature senescence via a p53-dependent mechanism [59]. Thus, CXCR2-binding chemokines can promote the arrest of cellular growth and eventually delay the early phase of tumorigenesis (Figure 5).

EMT is indispensable for embryogenesis [60]. EMT induces epithelial cells to lose the expression of components of cell polarity, such as E-cadherin, and reciprocally to express mesenchymal components of the cytoskeleton and to eventually acquire the motility and scattering properties. EMT is presumed to be associated with the capacity of tumor cells to invade and metastasize [60]. Prolonged exposure to TGF-*β* induces rat hepatocellular carcinoma cells to exhibit a mesenchymal phenotype, and a higher migratory and invasive capacity, coincident with increased CXCR4 expression, and these phenotypic changes are reduced by a CXCR4 antagonist [61]. Likewise, the expression of CXCL8 and its receptor, CXCR1, is induced in a human colon cancer cell line undergoing TGF-*β*-driven EMT [62]. EMT in cancer cells can be initiated by overexpression of several transcription factors, including Twist, Snail, and Brachyury [60]. Overexpression of Brachyury causes EMT in human pancreatic cancer cell lines, along with enhanced expression of CXCL8, CCL5, and CXCL1, while the inhibition of CXCL8 signaling pathway abrogates Brachyury-induced EMT phenotypes and invasive capacity of cells [63]. These observations suggest the crucial roles of several chemokines in EMT, an indispensable step for the malignant progression of cancer (Figure 5).

Chemokine receptor engagement can also activate the mitogen-activated protein (MAP)/Erk kinase pathway [64], leading to gene expression and cell proliferation (Figure 5). Human gastric cancer cell lines possess CXCR1 and CXCR2 and express epidermal growth factor (EGF) receptor, MMP-9, and VEGF, in response to CXCL8 [65]. The same pathway is utilized to promote proliferation of esophageal cancer cells [66] and melanoma cells [67].

In addition to the CXCL8 axis, several chemokines have impacts on proliferation and survival of tumor cells. CCR6 and CXCR6 promote the proliferation of colorectal cancer cells [68] and prostate cancer cells [69], respectively. Moreover, CCR10 activation in melanoma causes phosphatidylinositol-3-kinase- (PI3K-) mediated protection of tumor cells from apoptosis [70]. Similar observations are obtained on CCR7 activation in squamous cell carcinoma of the head and neck [71] (Figure 1). On the contrary, CCR5 blockade can enhance the proliferation of breast cancer cells, which express wild-type p53 [72]. Furthermore, disease-free survival is shorter in breast cancer patients bearing the CCR5Δ32 allele with a premature stop codon than in CCR5 wild-type patients; this holds true only in wild-type p53-expressing tumors [72]. Likewise, human hepatocellular carcinoma (HCC) cells express CCR1 and its ligands can affect the functions of human HCC cell lines [73].

CXCR4 is the most commonly detected chemokine receptor in tumor cells [38]. CXCR4 has an important role in the proliferation of various cancer cells including ovarian, glioma, melanoma, lung, renal, and thyroid cancer cells [38, 74]. The CXCL12/CXCR4 axis delivers surviving signals to hepatocellular carcinoma cells, ovarian carcinoma cells, and chronic leukemia cells, while CXCR4 blockade induces the apoptosis of these malignant cells [38, 61, 75] (Figure 5). CXCL12 expression correlates well with lower apoptosis in human myelodysplastic syndrome [76].

Chemokines can regulate the migration of tumor cells. CXCR4-expressing cells can migrate* in vitro* towards CXCL12 [77]. CCR7, CCR9, CXCR1, and CXCR2 are also detected in tumor cells and their ligands can induce the chemotaxis of the corresponding receptor-expressing cells [78, 79]. These chemokines can serve as inducers of invasion within the primary tumor and dissemination to distant organs (Figure 5). Moreover, human pancreatic ductal adenocarcinoma (PDAC) cells express CX3CR1 and CX3CL1 abundantly and the CX3CR1/CX3CL1 axis can regulate intraneural invasion of PDAC [80]. Adult T-cell leukemia (ATL) cells express frequently CCR4 and can migrate* in vitro* to CCL17 and CCL22, ligands for CCR4 [81]. The CCL17/CCL22-CCR4 axis may account for the frequent infiltration of ATL into skin and lymph nodes, where CCL17 and CCL22 are abundantly expressed.

Circulating tumor cells (CTCs) are presumed to be a source of metastasizing tumor cells, but they can also colonize at their original site [82]. This process, which is called as tumor self-seeding, can accelerate tumor growth and angiogenesis. CTCs produce ELR-motif-positive CXC chemokines including CXCL8 and CXCL1, and these chemokines eventually promote self-seeding [82].

Several models have been proposed to explain the molecular mechanisms underlying the enhancement and regulation of metastasis by the chemokines. Specific chemokine receptor-expressing tumor cells may migrate to organs with high expression levels of the corresponding chemokines along a concentration gradient [77]. This hypothesis may explain the tissue tropism observed in certain types of cancer, but there is little evidence to indicate the presence of chemokine concentration gradients between primary and metastatic sites. A transcellular CCR7 ligand gradient can be created when cancer cells produce CCR7 ligands under flow conditions and the resultant gradient can be the basis of lymphatic metastasis [83]. Thus, cancer cells themselves may actively promote their own metastasis and tropism by producing chemokines. Another plausible explanation is that the arrival of tumor cells in a specific organ is passive and that chemokine receptor expression provides tumor cells with an advantage to survive and grow in a different ligand-rich metastatic microenvironment [84]. Moreover, CXCL12 and a CCR7 ligand, CCL21, can induce the resistance of cancer cells to anoikis, which is a major hindrance to the metastatic spread of various types of cancer, by regulating proapoptotic Bmf and antiapoptotic Bcl-xL proteins [85]. Thus, chemokines may accelerate metastasis by promoting tumor cell proliferation or preventing tumor cell death.

Thus, chemokines can prevent tumorigenesis in the early phase by inducing cellular senescence while they can also promote invasion and metastasis by inducing EMT and enhancing the motility and survival of tumor cells (Figure 5).

## 4. Potential of Chemokine Targeting Therapy as Cancer Treatment

### 4.1. Chemokine-Mediated Enhancement in Tumor Immunity

**Figure 6:**
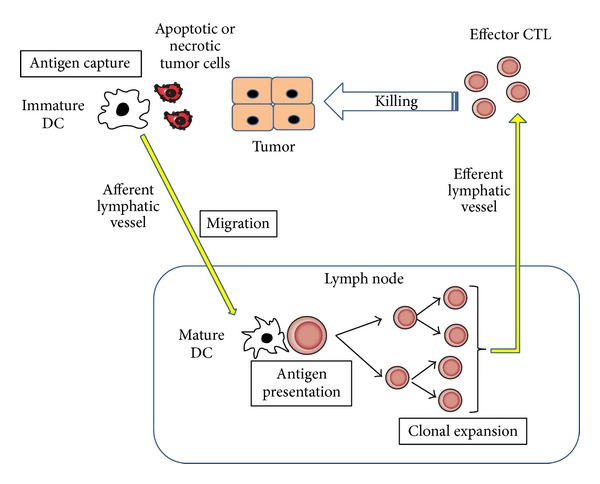
Tumor immunity generation process.

The establishment of tumor immunity is a multistage process: migration of DCs to tumor sites, capture of TAAs by DCs, migration of DCs to regional lymph nodes, antigen presentation to effector cells by DCs in regional lymph nodes, and migration of effector cells to tumor sites (Figure 6) [21, 22]. Chemokines have profound impacts on tumor immunity, particularly migration steps.

Immature dendritic cells move to the tumor tissues to phagocytose apoptotic tumor cells, capture tumor-associated antigens (TAAs), and migrate to draining lymph nodes, where DCs present antigens to induce specific CTLs [21–23]. Tumor-infiltrating DCs expressed CCR1 and CCR5, and a ligand for these receptors, CCL3, was abundantly detected in mouse bearing HCC [24]. Mirroring the capacity of CCL3 to mobilize CCR1- or CCR5-expressing immature DCs to peripheral blood from bone marrow [86], systemic administration of CCL3 increased the numbers of DCs in peripheral blood and tumor tissues and concomitantly augmented antitumor effects after radiofrequency ablation of murine HCCs [87]. Thus, CCL3 may be effective to enhance tumor immunity by attracting immature DCs to dying tumor cells.

CCL19 and CCL21, ligands for CCR7, can regulate DC migration to lymph nodes for antigen presentation to naïve T cells, which also utilize CCL19 and/or CCL21 to enter T-cell zone [88], and they can additionally attract NK cells to the lymph node. As a consequence, when CCL21 was injected into a regional lymph node of SV40-transgenic mice that developed bilateral multifocal lung adenocarcinomas, it increased CD4^+^ and CD8^+^ lymphocytes as well as DCs at lymph nodes and tumor sites and eventually led to a marked reduction in tumor burdens with enhanced survival [89]. Similar results were also obtained when CCL19 was injected intranodally into SV40-transgenic mice [90].

Low clinical efficacy of DC-based vaccines can be explained by a very limited capacity of* ex vivo* generated DC to move from the injected sites to draining lymph nodes [91]. In order to circumvent these problems, DCs are genetically modified to express CCL19 and CCL21. Indeed, intratumoral injection of* CCL21* gene-modified DCs resulted in more effective tumor growth inhibition than unmodified control DCs [92], together with intratumoral accumulation of DCs and T cells [93]. Moreover, even when* CCL21* gene-modified DCs were pulsed with tumor lysates and subsequently injected subcutaneously to tumor-free sites in tumor-bearing mice, it elicited a good antitumor response [92]. These promising preclinical results have led to ongoing phase I clinical trials [94].

Intratumoral administration of* CCL21* gene-modified DCs reduced a tumor burden in spontaneous murine lung carcinoma, accompanied with extensive T-cell infiltration and the enhanced IFN-*γ*, IL-12, CXCL9, and CXCL10 expression [95]. Moreover,* in vivo* depletion of either CXCL9 or CXCL10 significantly reduced the antitumor efficacy of* CCL21* gene-modified DCs, probably because CXCR3 is highly expressed by activated effector CD8^+^ T cells and Th1-type CD4^+^ T cells [96]. CXCL10 gene transduction into tumor cells had few effects on* in vitro* tumor cell proliferation but* in vivo* elicited a potent T-cell-dependent antitumor response [97]. Likewise, tumor cells expressing CXCL10 induced the infiltration of tumor-specific cytotoxic T cells into the tumor site [98]. Moreover, tumor cells induced these cytotoxic T cells to proliferate and to produce high level of IFN-*γ*, while CXCL10 expanded these tumor-specific T cells [98]. Similar observations were obtained on gene transduction of another ligand for CXCR3, CXCL11, into tumor cells [99]. Moreover, as T cells rapidly acquire CXCR3 expression upon activation with IL-2 [96], combined strategy of systemic IL-2 with intratumor CXCL9 administration was proven to be more efficacious than either cytokine alone, for augmenting tumor-associated immunity [26]. Thus, CXCR3-binding chemokines can be utilized to redirect the migration of effector T cells to tumor sites.

CCL2 was initially isolated as a factor which can also augment monocyte-mediated tumor cytostatic activity [100]. Indeed, tumor formation was suppressed* in vivo* but not* in vitro* when the tumor was genetically engineered to express* CCL2* gene [101]. CCL2-expressing cells elicited a predominantly monocytic infiltrate at the site of injection, suggesting the roles of infiltrating monocytes in tumor rejection process [101]. In another tumor models,* CCL2* gene transduction into tumor cells retarded tumor growth* in vivo* by inducing NK infiltration into tumor sites [102], because NK cells express CCR2, a receptor for CCL2 (Table 1). Moreover, NK cell infiltration was associated with elevated Th1 response in tumor sites [103], suggesting that CCL2 can regulate the infiltration and activation of Th1 cells in tumor sites by recruiting and activating NK cells.

Gene therapy using* CX3CL1* gene could activate T cells as well as NK cells to exert its antitumor responses [104–106]. Moreover, intratumoral injection of a DNA plasmid coding for a chimeric immunoglobulin combining with CX3CL1 chemokine domain provided strong antitumor activity [107]. The administration of this fusion protein with tumor antigens induced a strong* in vivo* antigen-specific T-cell proliferation and effector function, accompanied with myeloid DC accumulation [107]. Thus, CX3CL1 can redirect T cells and DCs as well as NK cells, thereby augmenting adaptive immunity to tumor antigens.

In order to enhance the capacity to move to tumor sites by utilizing the chemokine(s) produced by tumor cells, several groups genetically engineered T cells to express the corresponding chemokine receptor. The Reed-Sternberg cells of Hodgkin lymphoma predominantly produce CCL17 and CCL22 [108]. Effector CD8^+^ T cells lack CCR4, but CCR4 gene-modified CD8^+^ T cells migrated more efficiently to Hodgkin lymphoma site to inhibit tumor formation [109]. Similarly, CCL2 was highly secreted by malignant pleural mesothelioma cells, and CCR2 gene was further transduced to activate human T cells expressing a chimeric antibody receptor (CAR) directed to mesothelioma tumor antigen, mesothelin (mesoCAR T cells) [110]. The resultant gene-modified T cells exhibited enhanced antitumor responses accompanied with augmented T-cell infiltration into tumor sites, when they were given intravenously [110]. This novel gene therapy technology using a chemokine receptor can effectively enhance the migration of adoptively transferred T cells into tumor sites, where a corresponding chemokine is expressed abundantly.

### 4.2. Reversal of Suppressor Cell-Mediated Immune Suppression

Tumor immunity can frequently induce immune suppressive mechanisms to reduce the “immunity to self” by the action of several negative immunoregulatory receptors such as cytotoxic T lymphocyte antigen-4 (CTLA-4) and the programmed death receptor-1- (PD-1-) PD ligand-1 (PD-L1) axis. Consequently, the antagonizing monoclonal antibodies to CTLA-4, PD-1, or PD-L1 are effective against various types of cancer even at advanced stages [111, 112]. These observations indicate that reversal of immune suppression can be effective to enhance tumor immunity.

Tumor tissues contain the leukocytes that can diminish tumor immunity. The most predominant subset is TAMs [8, 12]. TAMs can promote tumor progression by inducing angiogenesis and suppression of adaptive and innate antitumor immunity (Figure 1). Circulating monocytes are mostly the precursor of these TAMs and are attracted into tumor sites, by several chemokines, particularly CCL2 [8, 12]. Indeed, systemic delivery of neutralizing anti-CCL2 antibody attenuated tumor burdens in human prostate cancer-bearing mice although its effects on TAMs have not been examined [113]. Moreover, CCL2 blockade reduced CCR2-expressing TAM infiltration and eventually tumor formation in chronic colitis-associated carcinogenesis [10]. Furthermore, CCL2 also recruited monocytes to pulmonary metastatic sites of murine breast cancer to promote the extravasation of tumor cells, a prerequisite step for metastasis, in monocyte-derived VEGF-dependent manner [11]. CCL2 blockade markedly reduced lung metastasis together with reduced monocyte/macrophage infiltration.

Another type of immune suppressor cells is MDSCs with a strong ability to suppress various T-cell functions [13]. CCL2 recruits MDSCs in several types of mouse cancer [14]. Moreover, CCL2-mediated MDSC accumulation can negatively regulate the entry of adoptively transferred activated CD8^+^ cells into tumor sites [114]. CCR2 deficiency, however, caused conversion of the MDSC phenotype to neutrophil lineage without affecting tumor growth [15], probably because MDSC contains a subset of immature neutrophils [115]. On the contrary, CXCR2 blockade reduced the infiltration of CXCR2-expressing granulocytic MDSCs and eventually tumor growth in chronic colitis-associated colon cancer [16].

Adult T-cell leukemia (ATL) cells are characterized by robust expression of CCR4 and can migrate* in vitro* to CCL17 and CCL22, ligands for CCR4 [81]. Humanized defucosylated monoclonal antibody to CCR4 has been obtained. The resultant antibody can exert more potent antibody-dependent cytotoxicity (ADCC) [116] and is capable of removing CCR4-expressing ATL cells in peripheral blood and bone marrow mainly by ADCC.

A large number of Treg cells often infiltrate into tumors and systemic removal of Treg cells enhances natural as well as vaccine-induced antitumor T-cell immunity [17]. Treg cells express CCR4 and its ligand, CCL22, mainly regulates intratumoral Treg infiltration in various tumors [17] (Figure 2). Indeed, intratumoral CCL22 expression correlated well with Foxp3 expression in colorectal carcinoma tissues [117]. In line with these observations, anti-CCR4 antibody treatment depletes Tregs and eventually evokes CD8^+^ T-cell response against TAAs [118]. Furthermore, CCL2 is also involved in Treg accumulation and, as a consequence, anti-CCL2 antibody augmented cancer immunotherapy against non-small cell lung cancer in mice with reduced intratumoral Tregs and increased numbers of intratumoral antigen-specific activated CD8^+^ cells, when it was administered in combination with a tumor vaccine [119]. These observations illustrate that targeting these chemokines can reduce intratumoral Treg cells, resulting in the enhancement of tumor immunity.

Recently, CCR1-expressing CD34^+^ immature myeloid cells have been detected in murine intestinal tumors with SMAD4 deficiency [120]. These cells expressed abundantly MMP-9 and MMP-2 and were involved in invasion. Moreover, a CCR1 antagonist suppressed colon cancer liver metastasis by blocking accumulation of CD34^+^ immature myeloid cells [121].

### 4.3. Other Strategies of Antitumor Therapy Targeting Chemokines

Neovascularization is crucial for tumor growth, progression, and metastasis [36]. The ELR motif-positive CXC chemokines, CXCL1, CXCL2, CXCL3, CXCL5, CXCL6, CXCL7, and CXCL8, can directly promote the migration and proliferation of endothelial cells and eventually neovascularization [37] (Figure 4). Indeed, the administration of anti-CXCL8 reduced the tumor sizes of human non-small cell lung cancer cells which are injected into severe combined immune deficient (SCID) mice in advance [122]. The reduction in tumor size was associated with a decline in tumor-associated vascular density and was accompanied by a decrease in spontaneous lung metastasis. CXCL12 [123] and three CC chemokines, CCL2, CCL11, and CCL16, have also been implicated in tumor neovascularization [39–41, 45]. However, it still remains elusive on the efficacy of targeting these chemokines for the control of tumor neovascularization.

CXCL4, CXCL9, CXCL10, and CXCL11 inhibit the angiogenesis induced by ELR motif-positive CXC chemokines, VEGF, and bFGF [42, 43]. Targeted expression of CXCL9 or intratumoral CXCL9 administration retarded* in vivo* tumor growth by inhibiting tumor-derived angiogenesis [26, 124]. Thus, these chemokines can be effective for tumor therapy by inhibiting neovascularization as well as inducing CXCR3-expressing cytotoxic T-cell infiltration.

## 5. Concluding Remarks

Cancer development and progression are profoundly affected by inflammatory and immune responses. Inflammatory responses consist of leukocyte infiltration, neovascularization, and fibrosis, while immune responses were exerted by immune cells such as lymphocytes and dendritic cells. Chemokines have great impacts on the cells involved in both inflammatory and immune responses. Moreover, several chemokines have direct effects on the proliferative and invasive properties of tumor cells. Consequently, chemokines play crucial roles in tumor development and progression by acting on cancerous and noncancerous cells. However, it is embarrassing that the same chemokine can induce tumor progression as well as protection against a tumor, in a context-dependent manner. Given the multifactorial roles of chemokines in carcinogenesis, the elucidation of their roles will further advance our understanding of the pathophysiological processes of tumor development and progression and will subsequently pave a novel way to develop a novel type of anticancer treatment by targeting chemokines.

## Figures and Tables

**Table 1:** The human chemokine system.

Chemokine receptor	Chemokines	Receptor expression in		
		Leukocytes	Epithelium	Endothelium
CXCR1	CXCL6, 8	PMN	+	−
CXCR2	CXCL1, 2, 3, 5, 6, 7, 8	PMN	+	+
CXCR3	CXCL4, 9, 10, 11	Th1, NK	−	+
CXCR4	CXCL12	Widespread	+	+
CXCR5	CXCL13	B	−	−
CXCR6	CXCL16	Activated T	+	−
CXCR7 (ACKR3)	CXCL12, CXCL11	Widespread	+	+
Unknown	CXCL14 (acts on monocytes)			
CCR1	CCL3, 4, 5, 7, 14, 15, 16, 23	Mo, Mϕ, iDC, NK	+	+
CCR2	CCL2, 7, 8, 12, 13	Mo, Mϕ, iDC, NK activated T, B	+	+
CCR3	CCL5, 7, 11, 13, 15, 24, 26, 28	Eo, Ba, Th2	−	+
CCR4	CCL2, 3, 5, 17, 22	iDC, Th2, NK, T, Mϕ	−	−
CCR5	CCL3, 4, 5, 8	Mo, Mϕ, NK, Th1 activated T	+	−
CCR6	CCL20	iDC, activated T, B	+	−
CCR7	CCL19, 21	mDC, Mϕ, naïve T activated T	+	−
CCR8	CCL1, 4, 17	Mo, iDC, Th2, Treg	−	−
CCR9	CCL25	T	+	−
CCR10	CCL27, 28	Activated T, Treg	+	−
Unknown	CCL18 (acts on mDC and naïve T)			
CX3CR1	CX3CL1	Mo, iDC, NK, Th1	+	−
XCR1	XCL1, 2	T, NK	−	−
Miscellaneous	Scavenger receptors for chemokines			
Duffy antigen (ACKR1)	CCL2, 5, 11, 13, 14			
	CXCL1, 2, 3, 7, 8			
D6 (ACKR2)	CCL2, 3, 4, 5, 7, 8, 12			
	CCL13, 14, 17, 22			
CCRRL1 (ACKR4)	CCL19, CCL21, CCL25			

Leukocyte anonyms are as follows. Ba: basophil, Eo: eosinophil, iDC: immature dendritic cell, mDC: mature dendritic cell, Mo: monocyte, Mϕ: macrophage, NK: natural killer cell, Th1: type I helper T cell, Th2: type II helper T cell, and Treg: regulatory T cell.
